# Study Protocol. ECSSIT – Elective Caesarean Section Syntocinon^® ^Infusion Trial. A multi-centre randomised controlled trial of oxytocin (Syntocinon^®^) 5 IU bolus and placebo infusion versus oxytocin 5 IU bolus and 40 IU infusion for the control of blood loss at elective caesarean section

**DOI:** 10.1186/1471-2393-9-36

**Published:** 2009-08-24

**Authors:** Deirdre J Murphy, Michael Carey, Alan A Montgomery, Sharon R Sheehan, The ECSSIT Study Group

**Affiliations:** 1 Obstetrics & Gynaecology, Coombe Women and Infants University Hospital, Trinity College Dublin, Dublin 8, Ireland; 2 Anaesthetics & Peri-operative Medicine, Coombe Women and Infants University Hospital, Dublin 8, Ireland; 3 Primary Care Research, Department of Community Based Medicine, University of Bristol, 25 Belgrave Road, Bristol, BS8 2AA, UK; 4 Coombe Women and Infants University Hospital, Trinity College Dublin, Dublin 8, Ireland

## Abstract

**Background:**

Caesarean section is one of the most commonly performed major operations in women throughout the world. Rates are escalating, with studies from the United States of America, the United Kingdom, China and the Republic of Ireland reporting rates between 20% and 25%. Operative morbidity includes haemorrhage, anaemia, blood transfusion and in severe cases, maternal death.

The value of routine oxytocics in the third stage of vaginal birth has been well established and it has been assumed that these benefits apply to caesarean delivery as well. A slow bolus dose of oxytocin is recommended following delivery of the baby at caesarean section. Some clinicians use an additional infusion of oxytocin for a further period following the procedure. Intravenous oxytocin has a very short half-life (4–10 minutes) therefore the potential advantage of an oxytocin infusion is that it maintains uterine contractility throughout the surgical procedure and immediate postpartum period, when most primary haemorrhages occur. The few trials to date addressing the optimal approach to preventing haemorrhage at caesarean section have been under-powered to evaluate clinically important outcomes. There has been no trial to date comparing the use of an intravenous slow bolus of oxytocin versus an oxytocin bolus and infusion.

**Methods and design:**

A multi-centre randomised controlled trial is proposed. The study will take place in five large maternity units in Ireland with collaboration between academics and clinicians in the disciplines of obstetrics and anaesthetics. It will involve 2000 women undergoing elective caesarean section after 36 weeks gestation. The main outcome measure will be major haemorrhage (blood loss >1000 ml). A study involving 2000 women will have 80% power to detect a 36% relative change in the risk of major haemorrhage with two-sided 5% alpha.

**Discussion:**

It is both important and timely that we evaluate the optimal approach to the management of the third stage at elective caesarean section. Safe operative delivery is now a priority and a reality for many pregnant women. Obstetricians, obstetric anaesthetists, midwives and pregnant women need high quality evidence on which to base management approaches. The overall aim is to reduce maternal haemorrhagic morbidity and its attendant risks at elective caesarean section.

**Trial registration:**

number: ISRCTN17813715

## Background

### Systematic review of literature

Caesarean section is one of the most commonly performed major operations in women throughout the world. Rates are escalating, with studies from the developed world reporting rates between 20% and 25%. [1-3] Rates vary considerably between centres within Ireland. [4]There are many possible approaches to performing a caesarean section, the aim being to achieve safe delivery of the infant with a minimum of maternal morbidity. Operative morbidity includes haemorrhage, anaemia, blood transfusion and the risks associated with receiving donor blood products. In severe cases it may result in hysterectomy, admission to an intensive care unit or death.

The active management of the third stage at caesarean section has received little attention to date. The value of routine oxytocics in the third stage of vaginal birth has been well established [5] and it has been assumed that these benefits apply to caesarean delivery as well. [6,7] The Royal College of Obstetricians and Gynaecologists (RCOG) Guideline on caesarean section recommends a slow intravenous bolus dose of 5 IU of oxytocin following delivery of the infant. [8] This is a lower dose than used previously by many obstetric anaesthetists (10–20 IU) [9] and is based on concerns about side effects such as maternal hypotension. [10] Conversely, a conservative approach to the use of oxytocin may increase the risk of haemorrhage.

Intravenous oxytocin has a very short half-life (4–10 minutes) therefore the potential advantage of an oxytocin infusion at caesarean section is that it maintains uterine contractility throughout the surgical procedure and immediate postpartum period, when most primary haemorrhages occur. Several randomised controlled trials have compared intravenous oxytocin with oral misoprostol, 15-methyl prostaglandin F2 alpha and carbetocin, reporting little evidence of benefit with the newer agents. [6,11-13] There has been no trial to date comparing the simpler approach of an intravenous slow bolus of oxytocin versus an oxytocin bolus and infusion. Both approaches are employed within current obstetric practice but there is great variation in the approach of individual clinicians and institution. There is very limited evidence to guide practice.

A search of Medline and Embase from 1965 to 2006 and of the Cochrane Library was undertaken, for relevant systematic reviews, meta-analyses, randomised controlled trials, and other clinical trials. The date of the last search was September 2006. We intend to update this as part of a Cochrane Systematic Review, but at the time of planning and starting the trial, there were no key changes since the search of September 2006. The main keywords used were: caesarean section, blood loss, haemorrhage, oxytocin, uterotonic agent, embolic agent, randomised controlled trial. In addition, when reviewing published reference lists, key articles cited were also retrieved and reviewed. The review of the literature has been divided into use of oxytocin at varying doses and comparative use of alternative uterotonic agents.

The literature relating to the use of oxytocin bolus doses at caesarean section is presented in Table 1. The current recommendation is to use a slow bolus of 5 IU intravenously after clamping of the cord. [8] Various approaches have been tested and it is possible that smaller doses (0.3 – 5 IU) may be sufficient for elective caesarean section. [14] Nonetheless we plan to use a 5 IU bolus of oxytocin within the trial in keeping with current recommended practice. Various alternative uterotonic agents have been evaluated in randomised controlled trials. (Table 2) Most published trials to date have had between 30 and 50 participants and have been under-powered to detect important clinical outcomes such as major haemorrhage and anaemia. The largest trial of over 500 women used "need for an additional uterotonic agent" as the primary outcome measure.

**Table 1:** Published studies evaluating doses of oxytocin (syntocinon)

Author, citation	Study design	Exposures	Outcome measures	Results	Conclusions
Sarna MC et al Anesth Analg 1997 84:753–6.	RCT 40 women Elective CS/regional	Oxytocin after cord clamping 5 IU vs 10 IU vs 15 IU vs 20 IU	Linear analogue of uterine tone EBL ΔHCT (pre-op/post-op)	No difference in any outcomes	No benefit in doses exceeding 5 IU
					
Zarzur Anesth Analg 1998 86:1334.	Dose-finding 20 women Elective CS	Oxytocin dosing	Uterine contractility Side effects	3 IU oxytocin Satisfactory No side effects	Low dose 3 IU sufficient
					
Carvalho JC et al Obstet Gynecol 2004 104:1005–10.	Dose-finding RCT single blind 40 women Elective CS/regional	Titrating increments of 0.5 IU oxytocin bolus Infusion 2.4 IU/hr	Minimum effective dose ED Uterine contraction EBL (calculated)	ED_90 _at 0.35 IU 100% at 1.0 IU	Low dose bolus < 5 IU effective
					
Balki M et al Obstet Gynecol 2006 107:45–50.	Dose-finding RCT single blind 30 women CS for labor arrest	Titrating increments of 0.5 IU oxytocin bolus Infusion 2.4 IU/hr	Minimum effective dose ED Uterine contraction EBL (calculated)	ED_90 _at 3.0 IU	Much higher dose than for elective CS Suggest alternative agent for non-elective

**Table 2:** Published studies comparing oxytocin (syntocinon) with alternative uterotonic agents

Author, citation	Study design	Exposures	Outcome measures	Results	Conclusions
Catanzarite VA et al Am J Perinatol 1990;7:39–42.	RCT double blind 46 women Elective CS	Oxytocin 20 IU iv bs PGF_2α_125 mcg im After placenta delivered	ΔHCT (EBL)	No difference in EBL	No benefit with carboprost
					
Chou MM et al Am J Obstet Gynecol 1994;171:1356–60.	RCT double blind 60 women Elective CS	Oxytocin 20 IU iv vs PGF_2α_125 mcg im	EBL ΔHCT, ΔHb Side effects (SE)	No significant differences	No benefit with carboprost
					
Boucher M et al J Perinatol 1998;18:202–7.	RCT double blind 57 women Elective CS	Oxytocin infusion 16 hr vs Carbetocin 100 mcg iv	EBL Side effects	Carbetocin as effective Lower EBL	Carbetocin as effective/reliable
					
Dansereau J et al Am J Obstet Gynecol 1999;180:670–6.	RCT double blind 635 women Elective CS	Oxytocin infusion 8 hr vs Carbetocin 100 mcg iv	Need for additional uterotonic agent	Carbetocin more effective 10% vs 4.7%	Carbetocin more effective than infusion only
					
Acharya G et al Acta Obst Gyn Scand 2001;80:245–50.	RCT single blind 60 women Elective CS/regional	Oxytocin 10 IU iv vs Misoprostol 400 mcg po	EBL, HCT, Hb, Side effects	No difference No SE	Misoprostol as safe/effective as 10 IU oxytocin
					
Lokugamage AU et al Aus NZ J Obst Gyne 2001;41:411–4.	RCT double blind 40 women Elective/Em CS	Oxytocin 10 IU iv vs Misoprostol 400 mcg po	EBL ΔHb Additional uterotonic	No difference	Misoprostol an alternative to 10 IU Need for large RCT

We propose to compare the simpler approach of oxytocin bolus versus oxytocin bolus and infusion. This will address the question whether an inexpensive and widely used drug can be employed in a more optimal way to prevent haemorrhage at caesarean section. We will be able to establish the number needed to treat to prevent one case of major haemorrhage with reasonable confidence limits.

The hypothesis is that an oxytocin infusion used in addition to an oxytocin bolus at elective caesarean section will reduce the risk of major haemorrhage and anaemia.

### National survey of current practice

Prior to undertaking this study it is important to establish current practice and the support for such a trial. A questionnaire was developed, composed of a series of closed answer questions with additional space for free text comments. The questionnaire focussed on the clinicians' use of oxytocin at caesarean section, perceived frequency of side effects, estimated average blood loss, rates of haemorrhage and willingness to participate in a future clinical trial. The questionnaire was sent to the lead consultant obstetrician and lead obstetric anaesthetist in all consultant-led obstetric units in the United Kingdom. Analysis was performed using SPSS Version 13.0 for windows. The findings of this survey are reported in detail in the European Journal of Obstetrics & Gynaecology and Reproductive Biology. [15]

Of the 434 questionnaires sent, 365 clinicians replied (response rate 84%), with none indicating that the questionnaire was inapplicable to their practice. Among the responders, 49% were lead obstetric consultants and 51% were lead obstetric anaesthetists. The various approaches to the use of oxytocin at the time of caesarean section are presented in Table 3. A slow bolus of 5 IU oxytocin was the preferred approach of obstetricians and anaesthetists (153, 86% and 171, 92% respectively). Routine oxytocin infusions were used by 72 clinicians (20%), and selective infusions were used for particular clinical situations by 289 (80%). Most clinicians used either 30 IU (158, 43%) or 40 IU (192, 53%) infusions over 4 hours, with a total of 38 different regimens. The perceived risk of side effects with oxytocin was low, particularly for infusions. Estimated "average" blood loss varied (150–1500 ml) with 56 clinicians (17%) and 93 (28%) reporting a >20% risk of postpartum haemorrhage for elective and emergency caesarean sections, respectively.

**Table 3:** Percentages refer to complete responses

	All n = 365 (%)	Obstetricians n = 179 (%)	Anaesthetists n = 186 (%)
Slow bolus 5 IU oxytocin	324 (89.5)	153 (86.4)	171 (92.4)
Slow bolus 10 IU oxytocin	44 (12.1)	28 (15.6)	16 (8.6)
Additional 5 IU bolus if clinically indicated	61 (17.0)	17 (9.5)	44 (23.5)
Routine use of oxytocin infusion	72 (19.8)	33 (18.4)	39 (21.1)
Selective use of oxytocin infusion	289 (79.8)	145 (81.0)	144 (78.7)
30 IU oxytocin infusion	158 (43.3)	83 (46.4)	76 (40.9)
40 IU oxytocin infusion	192 (53.3)	90 (50.8)	102 (55.0)

Approaches to the use of oxytocin at the time of caesarean section

This national survey of practice in the use of oxytocin for the prevention of blood loss at caesarean section found general consistency in the use of bolus oxytocin but a great deal of variation in the use of additional oxytocin infusions. The perceived risk of side effects with oxytocin was low, particularly for infusions. Obstetricians and anaesthetists reported a high rate of excess haemorrhage at caesarean section and more than half of the clinicians surveyed would participate in a randomised controlled trial comparing oxytocin bolus with bolus and infusion. There is wide variation in the approach to prevention of haemorrhage at caesarean section, reflecting a lack of robust evidence. This survey supports the need for further research into this important aspect of intrapartum care. The preferred approach was to use a 40 IU infusion of oxytocin in a solution of 0.9% saline in keeping with the recommendations of the Advanced Life Support in Obstetrics (ALSO) courses. This is the regimen we plan to use for the proposed trial.

The survey was carried out within the UK as the original plan (prior to relocation of the PI) was to conduct the study in Scotland. The practice of clinicians in Ireland is very similar to reported practice in the UK.

The hypothesis is that an oxytocin infusion used in addition to an oxytocin bolus at elective caesarean section will reduce the risk of major haemorrhage and anaemia.

### Aims and objectives

The aim of this study is to compare the effects of an intravenous slow bolus of oxytocin (5 IU) and placebo infusion (500 ml 0.9% saline) with an intravenous slow bolus of oxytocin (5 IU) and oxytocin infusion (40 IU in 500 ml 0.9% saline over 4 hours) at elective caesarean section in a double blind randomised trial.

Specific objectives are as follows:

### Primary outcomes

- to compare the incidence of major obstetric haemorrhage (≥ 1000 ml) following oxytocin bolus compared with oxytocin bolus and infusion

- to compare the need for an additional uterotonic agent between the two groups

Major obstetric haemorrhage will be defined by:

- measured blood loss as calculated from pre and post-operative haematocrit (see below)

### Secondary outcomes

- to compare the estimated mean operative blood loss between the two groups (as measured by theatre staff)

- to compare early lochial loss following oxytocin bolus compared with oxytocin bolus and infusion

- to compare the objective change in haemoglobin and haematocrit before and 48 hours after delivery between the two groups

- to compare the incidence of severe anaemia (Hb fall ≥ 20%) 48 hours after delivery between the two groups (or patient transfused).

- to compare the need for blood transfusion and/or blood products

- to compare the incidence of side effects (vomiting, hypotension)

- to compare the postnatal length of stay in theatre/recovery and in the hospital

### Cost-effectiveness

- to compare the cost-effectiveness of an oxytocin bolus regimen versus oxytocin bolus and infusion (separate protocol).

## Methods and design

### Recruitment and allocation

#### Inclusion criteria

The study will be limited to healthy women at term (> 36 weeks) with singleton pregnancies who are booked for elective caesarean section.

#### Exclusion criteria

Women with placenta praevia, thrombocytopenia, coagulopathies, a previous major obstetric haemorrhage (≥ 1000 ml), known fibroids or receiving anti-coagulant therapy will be excluded as will women who do not understand English and women under the age of 18 years. Other patients may be excluded at the clinician's discretion if a major haemorrhage is anticipated.

Women will be recruited to the trial in the third trimester when initial arrangements are being made for a booked elective caesarean section. The antenatal clinic midwife or research midwife/fellow will approach eligible women and offer information about the study. Women return on the day of surgery and spend a number of hours on the day ward or antenatal ward prior to surgery. At that point the woman's interest in participation in the study will be confirmed, an opportunity to ask additional questions will be provided and informed written consent will be completed. Randomisation will be performed using an automated web-based randomisation system hosted by the Bristol Randomised Trials Collaboration, a UKCRN-registered trials unit. Allocation will be stratified by centre and previous caesarean section (no/yes), and blocked using random permuted blocks of varying size.

The active or placebo infusion will be prepared by the research fellow/midwife. This will be done in the pharmacy department, away from theatre and will be labelled as "Trial drug – Placebo or 40 IU oxytocin". This will ensure blinding of everyone except the research fellow who makes up the infusion, and they will have no role in the management of the patient. Having the research fellow blinded is not possible for two reasons. Firstly, it is not possible to pre-prepare the infusions due to a lack of knowledge of the stability of oxytocin. Secondly, it has not been possible to obtain identically matched placebo ampoules.

### Intervention

Following written informed consent women will be randomised to receive either an intravenous slow bolus of oxytocin 5 IU and a placebo infusion (0.9% saline solution 500 ml over 4 hours), or oxytocin bolus 5 IU and oxytocin infusion (40 units in 500 ml 0.9% saline over 4 hours). Both the patient and the health care staff will be blinded to the intervention. The hospital pharmacist will produce numbered treatment kits containing either 1 × 5 IU ampoule or 1 × 5 IU ampoule and 4 × 10 IU ampoules. There will be labels and instructions for the research fellow to prepare the infusion so that the anaesthetist and other members of the investigator team remain blinded to the randomised treatment.

It is important that the surgical approach to caesarean section is standardised. Surgeons will be asked to operate to a standard procedure that specifies controlled cord traction for delivery of the placenta after administration of the oxytocin bolus, two layer closure of the uterine incision, and to avoid delivering the uterus for suturing unless clinically indicated. Deviations from the standard procedure will be recorded. Should the uterus remain atonic despite the trial intervention, the obstetrician/anaesthetist can use an additional uterotonic agent either replacing the trial infusion with a known infusion of oxytocin and/or use of a further agent.

Measurement of operative blood loss will be standardised. Disposable waterproof drapes are in use with pockets that capture all body fluids. The suction volume is measured. All swabs are weighed to measure additional blood loss. A more objective measure of blood loss will be calculated using pre- and post-operative haematocrit (see outcome measures below).

Anaesthesia will also be standardised. An intravenous bolus of 500 ml crystalloid should be given to all patients prior to spinal anaesthesia. A size 25G pencil-point needle should be used at a suitable lumbar interspace. The patient can be sitting or in the left lateral position for spinal anaesthesia. The anaesthetic solution will consist of 2 ml 0.5% hypertonic bupivocaine (2.2 ml in the sitting position), 10–20 μg fentanyl and 0.1 mg preservative free morphine. Anaesthesia should be to the level of T5, as assessed by touch. The patient will be tilted 15° to the left of supine and standard monitoring used as per the AAGBI guidelines. The sphygmomanometer cuff should be placed on the left arm. Haemodynamic monitoring will be performed before spinal anaesthesia, every minute until 10 minutes after delivery, every 15 minutes during the first postoperative hour then every 30 minutes for 4 hours. For the purpose of this study, baseline haemodynamic variables will be regarded as those immediately after clamping of the cord and before administration of syntocinon 5 IU. The investigators accept that individual anaesthetists may deviate from this protocol according to clinical needs. This is acceptable and should be documented on the case report form.

Intraoperative hypotension will be treated with intravenous boluses of phenylephrine 50 μg. If the heart rate falls below 60/min, intravenous boluses ephedrine 6 mg will be substituted. The anaesthetist will aim to maintain blood pressure at preoperative values. After delivery, the operating table tilt will be removed and the patient placed in the full supine position. Blood loss at operation will be replaced with colloid infusion or blood as deemed necessary by the anaesthetist. Postoperative pain relief will consist of regular non-steroidal analgesia combined with paracetamol. Opiates in the form of oxynorm can be used with caution considering the use of intrathecal opiates. Again it is acceptable for the local administrator to use any form of analgesia as per local practice and to document it on the case report form.

### Outcome measures

The primary outcome measures are major obstetric haemorrhage (estimated blood loss ≥ 1000 ml) and use of an additional uterotonic agent. Estimated blood loss of ≥1000 ml has been chosen to reflect major obstetric haemorrhage and it is widely accepted that clinicians underestimate rather than overestimate blood loss. This estimate will be based on the difference between pre-operative and post-operative haematocrit (EBL_calc _– Calculated estimated blood loss). [14]

The estimate is calculated as follows:



Where EBV estimated blood volume = Booking weight in kg × 85

Secondary outcomes include mean estimated blood loss (as measured by theatre staff), change in haemoglobin and haematocrit, significant anaemia (Hb fall = 20%) 48 hours after delivery, need for blood transfusion, side effects (including vomiting and hypotension) and length of postnatal stay in theatre/recovery and in hospital. Hypotension will be defined as a fall in blood pressure of more than 30% below the pre-operative blood pressure and/or use of ephedrine.

### Follow-up

A routine full blood count will be performed 48 hours after delivery to assess haemoglobin and haematocrit. Clinical follow-up of the mother will be completed prior to hospital discharge.

### Trial end

The trial will be considered complete after the final review of the last trial participant. The end of the trial will be notified to the Competent Authority and the Ethics Committee using the appropriate form.

### Statistical analysis

Data analysis and reporting will proceed according to CONSORT guidelines for randomised controlled trials, and will be conducted blinded to group status by the trial statistician and researcher. The first stage of analysis will be to use descriptive statistics to describe recruited individuals in relation to those eligible, and to investigate comparability of the trial arms at baseline. The primary analyses will involve intention-to-treat comparisons between the two groups for the primary outcomes, with transformation as appropriate after examination of distributions, and adjusted for stratification variables. Secondary analyses will investigate the effects of further adjustment for any variables displaying marked imbalance between the arms at baseline. Secondary outcomes will be analysed in a similar way. All analyses will use appropriate (that is, logistic or linear) regression models, with results presented as point estimates (odds ratios or difference in means), 95% confidence intervals and p values. Further secondary analyses will involve planned subgroup analyses and will use multivariable regression models with appropriate interaction terms to ascertain any differential effects in relation to repeat caesarean section, choice of anaesthetic, and operator experience.

### Pilot study results

We have completed an initial pilot study that has informed the study design allowing an estimate of appropriate sample size and likely recruitment, alongside an evaluation of outcome measures. The results of this study have been published by the European Journal of Obstetrics & Gynaecology and Reproductive Biology. [16] The pilot study represented a single centre randomised controlled trial of oxytocin 5 IU and placebo infusion versus oxytocin 5 IU bolus and oxytocin 30 IU infusion. We chose a 30 IU infusion as this was the approach recommended in the NICE Guideline on induction of labour. We have changed the study design to a 40 IU infusion reflecting preferred practice within the national survey and the recommendation of emergency obstetrics training courses (ALSO). The pilot results therefore reflect a slightly lower oxytocin concentration than is planned for the multi-centre study.

### Feasibility

**Figure 1:**
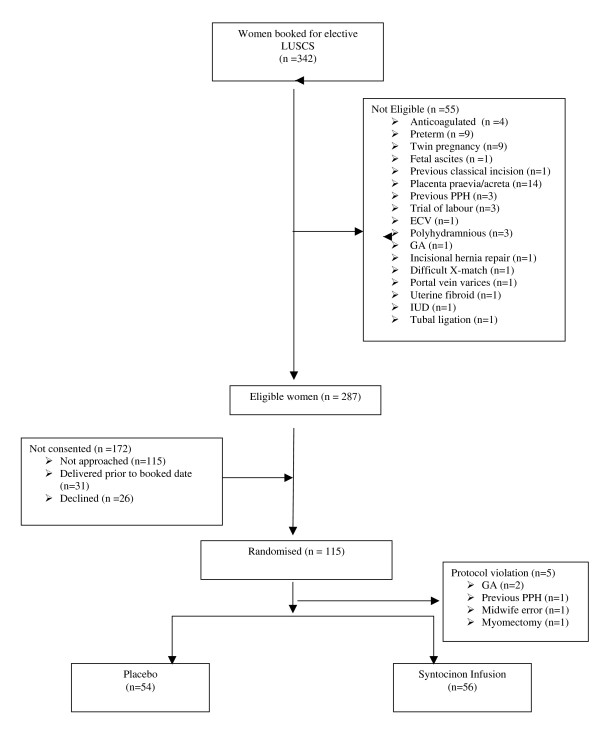
**Oxytocin at Caesarean Section Pilot Study – CONSORT Flowchart**.

The pilot study was conducted over an eight month period in a unit with 3400 deliveries a year. We received approval from the Edinburgh multi-centre research ethics committee (MREC) and approval of the UK MHRA in keeping with the requirements of the EU directive on Clinical Trials. The obstetricians, anaesthetists, midwives, theatre staff and hospital pharmacist were very supportive. We received a high level of support from women undergoing caesarean section, the majority of who were happy to participate in the trial. There were no serious unexpected adverse events. The recruitment is summarised in the Consort flow chart. (Figure 1)

### Sample size

We estimate from the pilot study that 5–10% of study participants will have an estimated blood loss of > 1000 ml. The rate is around 15% for the calculated estimate (EBL_calc_) and this is more likely to be an accurate reflection of actual blood loss. We found relative reductions of between 20–50% in the outcome measures on comparing infusion and placebo in the pilot study, but with wide 95% confidence intervals around these estimates. It is also possible that a pilot study will over or under estimate differences and we have therefore chosen conservative effect estimates for this multi-centre study.

With 1000 patients per group for analysis, the study will have 80% power at the 5% two-sided alpha level, to detect differences in the proportion of patients with clinician estimated blood loss >1000 ml of 15% vs. 10.7%, or 10% vs. 6.4%, equivalent to odds ratios of 0.62 and 0.68 respectively.

For the co-primary outcome of calculated blood loss, and estimated incidence in the control group of 14%, n = 1000 per group has 80% power to detect a difference 4.2%, equivalent to odds ratio of 0.67.

Based on the hospital statistics in the Dublin Maternity units for 2004 we estimate that there will be approximately 2500 elective caesarean sections at term each year between the five centres. Over the course of 30 months we would aim to recruit 2112 patients representing almost 34% of the available population (2112/6250) – this conservative estimate takes account of eligibility criteria and non-English speaking women. The sample size is slightly inflated from 2000 to allow for missing outcome data (full blood count not taken at the correct time or blood loss not measured).

### Timetable

Total 36 months: Commencing 01.09.07 – Estimated completion date 31.03.10

Month 0–2: Validate datasets; Raise study awareness; Finalise recruitment procedures

Month 3–33: Recruit participants and complete documentation and follow-up

Month 34–36: Analysis; Final report; Peer-review publications; Presentations.

### Governance issues

#### Ethical Committee Permission

Full ethical approval has previously been received for an eight- month local pilot study designed to develop the trial documentation and to test early feasibility issues.

05/MRE10/20 Approval date 16.03.05 MREC for Scotland

Ethical committee approval (Clinical Trials approved Committee) from the Coombe Women's Hospital, the National Maternity Hospital and the Rotunda Hospital, and approval of the Irish Medicines Board have been granted for this study.

IMB Application date 12.03.07 Approval date 27.04.07

Clinical trials approved committee Approval date 29.11.07

### Data management

Data will be collected in duplicate on a case report form (CRF) at the time of recruitment by a trained researcher. The researcher will also be responsible for ensuring that the details of the delivery are recorded and the blood loss measured. The inpatient notes will be marked so that they can easily be recovered following discharge from hospital. After discharge the CRF will be collected by the local co-ordinator and the completeness of the data checked against the woman's notes. Any errors will be followed up at this time. The data will be entered into a computer database (password protected) at the Coombe Women's Hospital.

### Trial Management Group (TMG)

This group will be in charge of the everyday running of the trial. The full group will meet 4-monthly and as required. Day-to-day decision making will be by Prof Murphy, Dr Michael Carey and the trial researcher.

### Trial Steering Committee

A trial steering committee will be set up which will have overall supervision of the trial. It will meet prior to commencement of the trial and then at least 6 monthly until completion. A meeting of the TSC will be held within a month of every DMEC meeting to consider their recommendations. An independent Chair will be sought for the TSC.

### Data monitoring and Ethics Committee (DMEC)

An independent safety and data monitoring committee will also be formed. They will meet yearly to examine recruitment figures, baseline data, retention and adverse events. The DMEC will not undertake any formal interim analyses for either safety or effectiveness and therefore there are no formal stopping rules. There is no evidence of adverse reactions associated with oxytocin infusion. Although adverse reactions have been shown to be associated with the 5 IU oxytocin bolus, all trial participants will receive a bolus, and thus we do not anticipate any differences in rates of adverse events between the groups. However all adverse events will be reported to the DMEC who will report these data to the Trial Steering Committee. Additionally, in the event of a postpartum haemorrhage, clinicians are permitted to discontinue the trial infusion and commence a known 40 IU oxytocin infusion.

### Safety considerations

Serious or unexpected serious adverse reactions (SUSARs) will be recorded and reported using the IMB approved SUSAR form. SUSARs include maternal death, surgery (other than caesarean section) in the 2 weeks following randomisation, transfusion of over 4 units of blood, admission to intensive care unit or suspected drug reactions. In the event of a SUSAR the form will be completed by the local trial co-ordinator and faxed to the trial co-ordinating centre at the Coombe Women's Hospital within 72 hours. From there the copies of the form will be sent to the trial statistician for unblinding and the chair of the Data Monitoring and Ethics Committee (DMEC). The IMB, the trial sponsor and the Chair of the MREC will also be informed by the DMEC Chair if considered appropriate.

## Discussion

### Potential and Implementation of the findings

It is both important and timely that we evaluate the optimal approach to the management of the third stage at elective caesarean section. The caesarean section rate has risen dramatically over the last two decades and continues to rise. Safe operative delivery is now a priority and a reality for many pregnant women. Obstetricians, obstetric anaesthetists, midwives and pregnant women need high quality evidence on which to base management approaches. The use of oxytocin in this context is a poorly evaluated component of obstetric care. The overall aim is to reduce maternal haemorrhagic morbidity and its attendant risks at elective caesarean section. In addition we will develop a protocol establishing the economic implications and patient perspective of the two approaches.

### Dissemination

We aim to raise awareness of this clinical question and our proposed research approach at both local and national meetings. A final report will be prepared for the funding body and papers will be prepared for peer-review publication and national/international dissemination.

## Competing interests

The authors declare that they have no competing interests.

## Authors' contributions

DM, MC, AM and SS drafted the manuscript, with input from other members of the ECSSIT Study Group.

## Pre-publication history

The pre-publication history for this paper can be accessed here:


